# The Impact of Antiviral Therapy and the Influence of Metabolic Cofactors on the Outcome of Chronic HCV Infection

**DOI:** 10.4061/2011/314301

**Published:** 2011-11-15

**Authors:** Marcello Persico, Savino Bruno, Andrea Costantino, Marta Mazza, Piero Luigi Almasio

**Affiliations:** ^1^Internal Medicine and Hepatology Division, Second University of Naples, Via Del Parco Carelli 36, 80123 Naples, Italy; ^2^Department of Internal Medicine, AO Fatebenefratelli e Oftalmico, Corso di Porta Nuova 23, 20121 Milano, Italy; ^3^Gastroenterology & Hepatology Unit, Di.Bi.M.I.S., University of Palermo, Piazza delle Cliniche, 2, 90127 Palermo, Italy

## Abstract

Natural history of HCV related chronic hepatitis is influenced and modified by many factors: virus features, coinfections and host characteristics. In particular, a peculiar genetic background of the host by conditioning the occurrence of intracellular metabolic derangements (i.e., insulin resistance) might contribute to accelerate the rate of progression to cirrhosis and eventually the occurrence of hepatocellular carcinoma (HCC) and death. Likely, direct interplays between virus genotype and host genetic background might be hypothesized at this level. Morbidity and mortality in cirrhosis is primarily associated with complications of liver cirrhosis (ascites, hepatic encephalopathy, jaundice, and gastroesophageal bleeding) and HCC occurrence. Therefore the main goal of therapy is to clear viral infection and decrease liver necro-inflammation that directly relates to development of cirrhosis and HCC. Among patients treated with Interferon-based therapy, those with sustained viral response showed a significant reduction of progression to cirrhosis and development of HCC. However, a residual risk of hepatocellular carcinoma still remains indicating the need for careful follow-up using ultrasonography every six months in cirrhotic patients, even in those showing persistently normal ALT and undetectable HCV RNA levels after antiviral therapy.

## 1. Introduction

**Figure 1:**
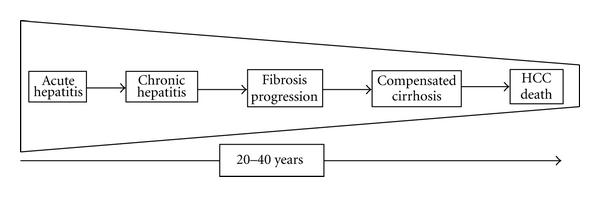
Natural history of chronic HCV infection.

The natural history of HCV infection is usually characterized by the transition from no perceivable acute to chronic infection, which may progress from a long-lasting asymptomatic condition up to a decompensated hepatic disease and/or hepatocellular carcinoma (HCC) development, which represents the main cause of liver-related death and liver transplantation in the Western World [1–5] (Figure 1). One hundred seventy million of people are infected worldwide, more than 30 million are affected by cirrhosis, and the incidence of HCC is about 1-2 million new cases/year. The actual estimated incidence is markedly decreased, and this is attributable to the employment of a safe transfusion's screening policy with a very high decrease number of new infections.

Recovery from acute infection is estimated to occur in 10–25% of patients, and the onset of the disease remains largely unknown since it is symptomless in the vast majority of cases. Moreover, since the availability of effective therapies has led to treat patients, the outcome and natural history of untreated people in order to analyse the events, their timing, and type of evolution has not been fully evaluated [6–12]. Furthermore, another essential phenomenon that has hampered the difficulty to obtain reliable information of the progression of the disease is because many cofactors can change the rate of progression of the disease. In particular, alcohol consumption together with other several factors (iron overload, hepatic steatosis, metabolic syndrome, viral coinfections) may play an important role in accelerating the progression of the disease [13].

A wide range of study designs (i.e., case-series, cross-sectional, case-control, cohort, clinical trial) has been developed in clinical investigation, and, among them, cohort studies have been largely employed. This type of studies represents a valid approach to by-pass the above-mentioned difficulties to assess the natural history of HCV infection. In fact, based on the statistical concept of “analysis by segments,” a typical approach for the cohort studies, the progression of HCV infection can be designed as the sequence of different, limited, and of presumably known duration stages of the disease. 

## 2. Natural History of HCV Infection

### 2.1. Studies Estimating Disease Progression

To define the natural history of an infectious disease, it is mandatory to establish the time of infection, to define the onset of the symptoms, to prospectively monitor expected end-points, to record the occurrence of risk factors (alcohol, other viruses, metabolic disorders, etc.), and to follow untreated patients for a long period of time. Unfortunately, although any possible HCV infection was demonstrable since 1989 [14], there is no hope to find such data in the studies published on this topic and a great heterogeneity among them might be mostly due to the diversity in the design of the studies themselves. In fact, in retrospective cohort studies, the presence of cirrhosis was ascertained in 17% up to 55% of chronic infected adults, and HCC in 1% up to 23% [15, 16]; on the other hand, in prospective cohort studies, the rate of progression to cirrhosis was of 7% to 16% and HCC of 0.7% to 1.3% per year [6, 17, 18]. In retrospective-prospective cohort studies, the prognosis of patients with chronic hepatitis C (CHC) was reported as benign and only a minority of the infected subjects ultimately developed cirrhosis [19, 20]. Similarly, Vogt and coworkers described a low rate of liver disease occurrence in children who underwent cardiac surgery before the implementations of the new rules for blood-donors screening [21]. By contrast, other studies reported a relative high rate of cirrhosis [22, 23], and, in particular, 5.9% of cirrhosis and liver-related death was found in a small cohort of anti-HCV US military recruits during a 45-year follow-up study [24]. 

### 2.2. Prognostic Factors of Disease Progression

A long-lasting elevated necroinflammatory activity seems to play a crucial role in the progression of the disease, and this clinical evidence seems to be supported also by data from patients with persistently normal ALT. Within this peculiar context, no progression of the disease was found at a second liver biopsy taken after 5 years of followup, with the exception of subjects in which the occurrence of flares-up seemed modifying the benign course of the disease [25–27]. However, even if the biochemistry is only partially indicative, no other reliable marker able to predict the progression of the disease has been identified so far. Moreover, the prognosis of subjects infected by HCV can also be affected by different variables, and many factors, either virus-related or host-related, have been investigated in order to understand the natural history of this infection. 

### 2.3. Viral Factors

Regardless the evidence that genotype 1b more than genotype 2 was reported to be associated with the development of HCC [28] and with a poor outcome of the disease [28–30], no data are available, so far, about other HCV genotypes. By contrast, while the viral load seems not to be associated with the histology activity index, it has relevant implications rather with viral response to therapy [31–34]. The presence of other viral coinfections (i.e., HIV, HBV) speeds up the clinical course of the disease [35, 36]. The coinfection with HBV is most common in several high-risk populations, such as injection drug users (IDU) [37, 38], patients on hemodialysis [39], patients undergoing organ transplantation [40], HIV-positive individuals [41, 42], and *β*-thalassemia patients [43, 44]. In all these categories, liver injury was more severe in terms of progression of fibrosis and liver cirrhosis, hepatic decompensation [45–50]. Moreover in this subgroup has been shown an increased risk of developing HCC [51–53]. Also, the presence of HIV has a negative impact on the natural history of HCV since the progression to cirrhosis is higher in coinfected [54, 55]. HCV/HIV coinfection is associated to develop other complications, such as hematologic disorders [56, 57], kidney disease [58, 59], cardiovascular disease [60, 61], and neurologic status [62–65]. 

### 2.4. Host Factors

Among the host-related factors, female gender and young age seem to influence the outcome of CHC [66]. In the last few years, another important aspect represented by the presence of comorbidities was shown to impact on the evolution of chronic HCV infection. Hepatic steatosis, obesity, and/or an insulin resistance must be considered important determinants of the disease progression [67–70]. Recent data also show that genotype 1 might specifically interfere with a peculiar host genetic background conditioning an altered intracellular insulin signalling [71, 72]. However, the relationship between body mass, insulin resistance, steatosis, and clinical outcomes is still complex. In study of long-term peginterferon treatment (HALT-C), the modifiable risk factors for liver disease progression were studied, and insulin resistance was strongly associated with outcomes [73]. On the other hand, the significance of both iron overload and “occult” HBV infection remains still controversial [74–77]. The iron overload plays an important role. The complexity of the interplay between iron and CHC is underscored by recent finding that mutations in *HFE*, particularly the common H63D variation, while being associated with higher hepatic and total body iron, is also related to a significantly higher likelihood of complete and sustained responses to antiviral therapy [78]. A recent study shows that stainable iron in hepatocytes and portal tract cells is a predictor of progression and clinical and histological outcomes in advanced chronic hepatitis C while chronic administration low-dose of peginterferon did not improve outcomes, nor iron variables [79].

### 2.5. External Factors

Among investigated cofactors, the alcohol abuse is the most important one which can dramatically change the course of the disease. Persistent intake even of low alcohol amount may seriously interfere with the progression of the disease [80]. Alcohol consumption, even at low daily intake, has been proposed as a risk factor for the progression of liver disease in chronic HCV infection [81, 82]. Alcohol consumption has a very significant impact on both the histologic and clinical progression of chronic HCV infection. The mechanisms whereby alcohol enhances HCV infection and histologic damage are still unclear [83].

Geographical differences in the evolution of chronic HCV infection to cirrhosis should also be considered: in USA, and Europe, this percentage is almost 15% (range 8–24%), but in Japan it ranges between 30% and 46%. Similarly, the percentage of evolution to HCC is 0.7–1.3% in Western countries, but significantly higher in the Far East and Japan where the rate ranges from 10% to 19% [84].

## 3. The Natural Course of HCV-Induced Cirrhosis

The natural history of HCV-induced chronic liver disease remains poorly measurable. As discussed above, a practical approach used to by-pass the above-mentioned difficulties is represented by the “analysis by segments” [85–88]. All these studies agreed that the development of HCC represents the main cause of death in cirrhotic patients and male sex, older age, high alpha-fetoprotein levels, and HBV coinfection were considered the main risk factors of development of the tumor. Interestingly, some studies have also described as risk of HCC occurrence the presence of oesophageal varices [29, 89]. The presence of gastrooesophageal varices is commonly considered an important risk factor of hepatic decompensation, and certainly they are expression of portal hypertension. Based on this assumption, the hypothesis that explains the association between varices and HCC development is based on the assumption that an impaired regional blood flow due to portal hypertension might determine local hypoxia which stimulates the synthesis of angiogenic factors [90]. Moreover, HCV genotype 1b was recently confirmed as a risk factor associated with HCC development in a prospective study [91, 92]. Accordingly, a biological plausibility has been recently provided showing a possible interplay by HCV and a cellular oncosuppressor determining a linkage between a mutated viral site and a different sensitive to damage-induced apoptosis [93]. 

Taking into account all the available information, patterns of progression to cirrhosis are very different: fast if it occurs in less than 20 years, intermediate in 20–50 years, slow in more than 50 years. Moreover, in some patients, there is no progression at all. The causes of this divergence in outcome are still poorly understood. However, survival at 5 years is reduced to 50% after an episode of decompensation [94–96].

Future studies on the natural history of HCV disease should be aimed to identify patients with high risk of disease progression and to assess the impact of therapy on disease outcome. 

## 4. The Impact of Antiviral Therapy on Natural Course of the Disease

The lack of randomised studies hampers a reliable estimation of the impact of antiviral treatment on the long-term outcome of patients with HCV infection. Several cohort studies, designed to assess the response to Interferon (IFN) therapy in cirrhotic patients, documented a better prognosis for patients who received IFN regardless of HCV-RNA eradication. Two retrospective reports [97, 98] confirmed these data, and altogether these studies have demonstrated that sustained virological response (SVR) is significantly associated with a reduction of decompensation rate, HCC occurrence, and liver-related mortality. Therefore, liver disease morbidity and mortality is dependent on successful antiviral therapy, and peginterferon plus ribavirin is, at present time, the treatment of choice before direct-acting antivirals (DAAs) will be available [99]. 

### 4.1. Progression from Chronic Hepatitis C to Cirrhosis 

The assessment of histological activity and fibrosis stage of liver biopsies performed before and after antiviral treatment remains the most reliable parameter to evaluate short-term benefit of viral clearance. In 487 Japanese patients who underwent paired liver biopsy, samples obtained from 1 to 10 years apart showed a regression of fibrosis related to SVR [100]. Accordingly, other European studies by analyzing the impact of SVR on the long-term clinical outcome of chronic hepatitis C concluded that SVR was significantly associated with a decrease in fibrosis score [11, 101–103]. More recently, George et al. demonstrated in a large cohort that clinical, virologic, biochemical, and histological outcomes of patients followed up to 5 years after SVR are favourable, and recovery of normal or nearly normal liver architecture is possible [104].

The available literature on the likelihood of progression to cirrhosis in treated patients is limited [105–117]. Once SVR is achieved the histological progression is uncommon (7 out of 1,662 treated patients with SVR) (Table 1). On the contrary, in relapsers or nonresponder patients, the rate of disease evolution to cirrhosis is much more frequent (162 out of 2,078).

### 4.2. Progression from Compensated to Decompensated Cirrhosis and Hepatocellular Carcinoma

Unsatisfactory SVR rates have been attained in patients with cirrhosis when using traditional monotherapy regimens of recombinant IFN alfa or peginterferon [118], and all clinical trials for drug registration considered decompensated liver disease as an exclusion criterion [119–122]. In these studies, SVR rates were calculated by pooling together patients with different degree of hepatic fibrosis, ranging from marginal bridging fibrosis to compensated cirrhosis. As a consequence, no definite conclusion could be reached about the efficacy and safety of treatment in this cohort of patients. Nevertheless, results emerging from these studies suggested that treatment of patients with well-compensated HCV-induced cirrhosis using PEG-IFN alfa-2b plus RBV may be considered a reliable option in “easy to treat” “genotypes” (2a/c, 3a), while in the “difficult to treat” ones (1a, 1b, and 4 genotype), the results are not satisfactory. In this subgroup of patients, the use of baseline and on-treatment predictors of response may enhance virologic outcomes and refinement of the regimens investigated is warranted. Further studies are also needed to develop treatment schedules for individual genotype populations. 

## 5. Summary

Natural history of HCV-related chronic hepatitis is conditioned by virus features, coinfections, and host characteristics. In particular, a peculiar genetic background of the host by conditioning the occurrence of intracellular metabolic derangements (i.e., insulin resistance) might contribute to accelerate the rate of progression to cirrhosis and eventually the occurrence of HCC. Likely, direct interplays between virus genotype and host genetic background might be hypothesized at this level. 

Morbidity and mortality in cirrhosis is primarily associated with complications of liver cirrhosis and HCC occurrence. Therefore, the main goal of therapy is to inhibit viral replication and decrease liver necroinflammation that directly relates to development of cirrhosis and HCC. 

Among patients treated with IFN-based therapy, those with SVR showed a significant reduction of progression to cirrhosis and development of HCC. However, a residual risk of hepatocellular carcinoma still remains indicating the need for careful followup using ultrasonography every six months in cirrhotic patients, even in those showing persistently normal ALT and undetectable HCV RNA levels after antiviral therapy.

## Figures and Tables

**Table 1:** Cumulative rate of development of cirrhosis in patients with HCV-related chronic hepatitis according to sustained viral response (SVR); n/a: not available.

Author	Reference	Treated patients	Followup (years)	Rate of cirrhosis after SVR	Rate of cirrhosis in absence of SVR
Marcellin et al.	[105]	450	1–7.6	0/75	n/a
Sata et al.	[106]	63	0.6–3.8	0/25	9/38 (23.7%)
Lau et al.	[107]	10	10–13	0/5	2/5 (40.0%)
Cammà et al.	[108]	62	0.7–9	0/62	5/360 (1.3%)
Ajello et al.	[109]	31	10	1/10 (10.0%)	n/a
Morisco et al.	[110]	191	4	0/39	12/115 (10.4%)
Gallego et al.	[111]	79	4	0/11	33/87 (37.9%)
Giannini et al.	[112]	36	1–6	0/15	3/21 (14.3%)
Shindo et al.	[113]	250	8–11	0/67	62/183 (33.9%)
Swain et al.	[114]	997	8	0/989	8/997 (0.8%)
Veldt et al.	[115]	343	1.6	6/110 (5.5%)	3/15 (20%)
Ciancio et al.	[116]	97	7	0/83	3/86 (3.5%)
Chavalitdhamrong and Tanwandee	[117]	n/a	3	0/171	27/171 (15.8%)

				**7/1,662 (0.42%)**	**162/2,078 (7.8%)**
